# Difficulties Experienced by Patients with Knee Osteoarthritis during the Conservative Treatment Process: A Qualitative Study

**DOI:** 10.3390/jcm12206523

**Published:** 2023-10-14

**Authors:** Seçkin Özcan, Hakan Yurten

**Affiliations:** 1 Department of Orthopedics and Traumatology, Yalova Education and Research Hospital, 77100 Yalova, Turkey; 2 Department of Orthopedics and Traumatology, Elazığ Fethi Sekin City Hospital, 23100 Elazığ, Turkey; hakanyurten@gmail.com

**Keywords:** knee osteoarthritis, conservative treatment, qualitative study, compliance with treatment

## Abstract

Background and Objectives: This study aimed to investigate the difficulties faced by patients with knee osteoarthritis during the conservative treatment process. Materials and Methods: We included twenty-one patients who were diagnosed with knee osteoarthritis and admitted to the orthopedics and traumatology outpatient clinic of the hospital where the researcher worked between January 2022 and April 2022. We interviewed each patient using semi-structured face-to-face interviews. To analyze the interviews, the researcher used the directed content analysis method. Data were analyzed using the NVIVO 10 software package. The authors and the expert trained in qualitative research who generously supported the authors continued the analysis independently of each other until they reached a consensus. Results: After analysis of the interviews held with the participants, the following three main themes emerged: lack of information about conservative treatment, frequent change of physicians, and non-compliance with lifestyle changes. Two sub-themes were identified within the theme of frequent change of physicians: distrusting health personnel, and not being able to make an appointment. In addition, most of the patients were not knowledgeable enough about either the definition of the disease or the treatment process. These patients stated that they were confused because they had to change physicians frequently; thus, they distrusted physicians because each physician they visited made a different plan for the treatment process. Conclusions: At the end of the study, we determined that during the conservative treatment process of patients with knee osteoarthritis, a multidisciplinary approach should be adopted, and orthopedic surgeons, physical therapy and rehabilitation physicians, dietitians, and physiotherapists should be in harmony with the patient. In addition, health personnel should provide patients with detailed information to eliminate questions they have during the conservative treatment process. In order for healthcare team members to establish a trusting relationship between the patients, they should allocate enough time to the patient.

## 1. Introduction

Osteoarthritis is the most common degenerative joint disease affecting sufferers’ daily lives worldwide [1]. The prevalence of osteoarthritis is increasing day by day as the population ages and the prevalence of obesity increases [2,3]. Knee osteoarthritis is the most common form of osteoarthritis and a leading cause of pain and disability [4]. Approximately 40% of people over the age of 65 suffer from symptomatic knee osteoarthritis [5], which makes knee osteoarthritis a significant burden on the healthcare system [6]. The prevention and management of knee osteoarthritis is of global concern [1]. There is currently no medication to cure osteoarthritis [7]. Treatment planning of knee osteoarthritis is made according to clinical symptoms and radiological grading. Conservative treatment is useful for patients with Kellgren and Lawrence’s (KL) grade 1–3, which are early stages of osteoarthritis. Higher grades indicate more severe signs of osteoarthritis. Knee replacement surgery is only suitable for symptomatic patients with advanced-stage (KL grade 4) osteoarthritis, and is not recommended for approximately 80% of patients [8,9].

Currently, the focus is on strategies to prevent or delay surgical interventions in the early stages of the disease [8]. In current guidelines, primary treatment recommendation for knee osteoarthritis is conservative (non-surgical) treatments [10,11,12], a combination of pharmacological and non-pharmacological treatments [13,14]. Therefore, using analgesics and non-steroidal anti-inflammatory drugs (NSAIDs) informing patients and encouraging them to exercise and lose weight are the main methods implemented. NSAIDs are frequently preferred to relieve pain and inflammation. However, the prolonged intake of these drugs may cause undesired side effects. Therefore, the use of supplements such as the glycosaminoglycan, chondroitin sulfate can be considered as a useful support or therapeutic treatments in low-grade patients [10,11,12]. In several studies, conservative treatment methods have been demonstrated to improve the quality of life of people with knee osteoarthritis, to delay surgery, and to reduce costs on the healthcare system [8,15,16]. However, it is difficult to routinely incorporate conservative treatment methods into the care of patients [9]. As was indicated in a study, the patients with knee osteoarthritis had poor compliance with treatment [7] and they were reluctant to undergo physiotherapy [17]. In a study conducted in Norway, the rate of the patients who adhered to non-pharmacological conservative treatment methods was 44% [18]. Although there are qualitative studies in the literature aimed at determining patients’ knowledge levels [19], their perspectives [20,21] and needs [22] regarding conservative treatment, no studies have been conducted on this subject in Turkey. Patients’ perception of the disease and difficulties they experience during the disease process may be affected by sociocultural differences [22]. Although understanding difficulties experienced by patients during the conservative treatment of knee osteoarthritis is of great importance, this issue has not been adequately investigated in Turkey. Therefore, in the present study, we aimed to investigate difficulties faced by patients with knee osteoarthritis during the conservative treatment process.

## 2. Materials and Methods

### 2.1. Study Design

In our study, we used phenomenological design, one of the qualitative research methods, which is recommended to comprehensively investigate condition-specific factors and the effects of these factors on individuals. We used the Consolidated Criteria for Reporting Qualitative research (COREQ) checklist to ensure comprehensive reporting of our study [23].

### 2.2. Setting and Participants

Patients who were diagnosed with knee osteoarthritis and presented to the orthopedics and traumatology outpatient clinic of the hospital where the researcher worked between January 2022 and April 2022 were included in this study. To select eligible patients, the researcher screened the electronic health records of the patients. When the researcher scanned the electronic health records in a public hospital in western Turkey between the specified dates, he accessed the records of 55 patients diagnosed with knee osteoarthritis. Of the screened patients those who met the following inclusion criteria were included in the study: (1) having a clinical and/or radiological diagnosis of knee osteoarthritis, (2) not having a history of inflammatory arthritis, (3) not having had a knee surgery before, (4) not having a cognitive or communication impairment. All patients were invited to the outpatient clinic after being given information about the study via phone call. In order not to cause any bias among the participants, they were called by the secretary working in the orthopedics and traumatology outpatient clinic. Of the patients called, 15 declined to participate in the study, 7 were not included in the study because they had a history of inflammatory arthritis, and 2 were not included in the study because they could not communicate in Turkish. Thus, we included a total of 31 patients in our study.

### 2.3. Data Collection

In this qualitative study, the physician who has been working as a specialist in the field of orthopedics and traumatology for 10 years and who is well trained in communicating with patients conducted semi-structured face-to-face interviews. The physician maintained the interviews with the identified patients until data saturation was reached. As stated in the literature, if no different ideas emerge from three consecutive interviews, then data saturation is considered to be reached [24]. For the 21st patient, it was decided that the data reached saturation (i.e., no new information was obtained, repeated information was obtained, and no further coding was available), and the interviews were terminated.

The researcher used the following two forms as data collection tools: the “Descriptive Information Form”, which questions the participants’ characteristics such as age, sex, stage of the disease, and conservative treatment method they undergo, and the semi-structured “Interview Form”, developed based on previous studies on knee osteoarthritis [3,7,22] and international guidelines. In the Interview Form, the following points are questioned: the process experienced by patients with knee osteoarthritis from diagnosis to treatment, the information they have about their diseases, treatment methods they are recommended to undergo, and follow-up of their diseases. In the form, the patients were asked open-ended questions to understand the issues investigated adequately (Table 1). A calm and quiet environment was created for the patients to respond to the questions without prejudice. The interview with each patient lasted an average of 53 (min: 28–max: 78) minutes.

### 2.4. Data Analysis

All the interviews were audio-recorded and then transcribed verbatim. To analyze the interviews, the researcher used the directed content analysis method. The researcher received support from an expert trained in qualitative research who reviewed all the interviews independently. The researcher analyzed the data using the NVIVO 10 software. He clustered the words representing the same field. During the coding process, he defined new codes for each new idea, and defined new codes for ideas that could not be copied into predetermined areas. The researcher and the expert continued the analysis independently of each other until they reached a consensus.

### 2.5. Ethical Approval

This study was performed under the approval of our institution’s ethical review board (Document number: 2022/118) and was conducted under the Declaration of Helsinki. Informed consent was obtained from each participant before the interview.

## 3. Results

Of the 21 participants, 13 were women. Their mean age was 63.09 (Min: 56, Max: 70) years. Most of the patients were homemakers and primary school graduates. The demographic data and clinical characteristics of the patients, such as body mass index, side, and KL grade, are presented in Table 2. When the researcher scanned the electronic health records in a public hospital in western Turkey between the specified dates, he accessed the records of 55 patients diagnosed with knee osteoarthritis. Of them, 15 declined to participate in the study, 7 were not included in the study because they had a history of inflammatory arthritis, and 2 were not included in the study because they could not communicate in Turkish.

After the analysis of the interviews held with the participants, the following three main themes emerged: Lack of information about conservative treatment and frequent change of physicians and noncompliance with lifestyle changes (Table 3). The participants generally mentioned that they had difficulty in accessing healthcare services and continuing the conservative treatment process. The problems experienced by the patients while they accessed health services affected their compliance with the treatment process adversely. In addition, most of the patients stated that they were not knowledgeable enough about either the definition of the disease or the treatment process. When the researcher analyzed the interviews, he also realized that knowledge of those who stated they were knowledgeable was not correct. The patients who lacked knowledge stated that they were confused because approaches displayed by the physicians during the conservative treatment process varied from one physician to another. They also stated that they were confused because they had to change physicians frequently and that they felt distrustful of the physicians because each physician they visited made a different plan for the conservative treatment process.

### 3.1. Theme 1: Lack of Knowledge about Conservative Treatments

Of the patients, while some lacked knowledge, some had inadequate or incorrect knowledge about their disease and the conservative treatment process. They generally considered the cause of their illnesses to be old age. Most of them thought that they could only be treated with medication. When the researcher asked the patients, who said they had consulted many doctors how much they knew about their disease, most of them described it as follows: ‘calcification’, ‘osteoporosis’, and ‘loss of fluid, which suggests that they needed more information about their disease and treatment. The researcher observed that most of the patients who expressed their opinions about conservative treatment methods did not adhere to these treatments correctly and effectively because they did not have enough knowledge about the recommended treatment methods.

“Because I am old, my knees have run out of fluid, my bones have become thinner, I have no idea about the treatment.”

(Participant (P)-16)

“I think my knees hurt because I’m overweight. So, I have to lose weight, but I can’t because I can’t walk due to the pain in my knees.”

(P-8)

“I worked hard when I was young. I never cared myself. That’s why my knees are like this. Now, its treatment is very difficult, medication does not work.”

(P-20)

“I have severe pain in my knees, but I don’t know why, I don’t know how to relieve this pain.”

(P-13)

“I have to take painkillers constantly to relieve the pain in my knee; it does not go away otherwise. I don’t know if there is a treatment other than medication.”

(P-6)

“I know I have calcification in my knees. My knee pain started suddenly. I didn’t fall, I didn’t hit my knee, but I don’t know why I have pain.”

(P-21)

“Actually, I want to know a lot about my disease, but I do not know how I can learn. When I go to the physician to be examined, I usually cannot ask questions because there are many patients waiting at the physician’s door.”

(P-12)

I wonder if there is treatment for my disease other than surgery. “I don’t want to undergo surgery, but I’m not sure if the pain in my knee will go away without surgery.”

(P-19)

### 3.2. Theme 2: Frequent Physician Change

One of the leading obstacles to compliance with the conservative treatment process was that the patients had to change physicians frequently. Of the patients, those whose complaints continued after the use of the medication stated that they changed physicians because they wanted to be followed by another physician or because they could not reach the same physician again. Most of the patients stated that they were constantly examined by different physicians and that physicians gave them different suggestions during the conservative treatment process. Distrusting health personnel and not being able to make an appointment emerged as the sub-themes of the frequent physician change.

#### 3.2.1. Subtheme 2.1: Distrusting Health Personnel

The researcher observed that the patients distrusted the treatment administered by the physician. They stated that they were confused due to different recommendations made by each physician during the conservative treatment process, so they had to change physicians frequently. Most of the patients who were confused were negatively affected by the conservative treatment process and had poor compliance with the treatment. The patients stated that physicians should allocate more time to them if a trusted relationship is to be established between the patient and physician, and that the current healthcare system is not effective enough to cure them. We observed that high patient density prevented physicians from allocating enough time to each patient, which negatively affected the patient–physician relationship.

“The first physician I consulted because of the pain in my knees prescribed medicine. When I visited another doctor when my medication was finished, he suggested that I be treated with surgery. I will ask another doctor.”

(P-15)

“My physician at the state hospital referred me to the physical therapy department. However, when I visited another doctor at a private hospital, he suggested an injection into my knee before physical therapy. Because I’m confused, I want to ask another doctor.”

(P-14)

“I have been taking medication for a long time. My physician said that I would be better if I had physical therapy and medication together. However, I never went to that doctor again because I didn’t think physical therapy would help.”

(P-19)

“When I visited the doctor to be examined, I waited in line for a long time. It was so crowded in front of the doctor’s door that I wondered how he could spare time for so many patients. Really, most of the questions in my mind remained unanswered. I will go and try to ask another doctor these questions.”

(P-21)

“Actually, the doctor should inform us about our treatment in detail. He prescribed me medication and said that the pain in my knee would go away with this medication. If the pain doesn’t go away, he advised me to go to a physiatrist. I have already been using these medications for years. I don’t think physical therapy will work either. Why should I go to this doctor again?”

(P-16)

#### 3.2.2. Subtheme 2.2: Not Being Able to Make an Appointment

Most of the patients stated that they had difficulty in continuing their follow-up in the conservative treatment process. The leading reason was that they could not make an appointment with the same physician who prescribed the follow-up-treatment process due to the hospital density. They also had difficulty accessing both orthopedists and physiatrists. Almost all of the patients were unable to keep up with the follow-up-treatment processes because they could not make an appointment for months. In addition, patients who were deemed suitable to start a physiotherapy program could not ensure the continuity of their treatment due to the density in the physical therapy units. Thus, the patients had difficulties both in the pharmacological treatment process and in the physical therapy and rehabilitation process. This suggests that the patients who had problems accessing health services went through a period without treatment for a long time, and then did not keep up with the treatment steps other than taking oral medication and with regular follow-up protocols.

“The physician told me to come for a check-up if my complaints did not improve after taking my medications. However, I had to wait after my medication was finished because I could not make an appointment. The earliest appointment I could make was 1.5 months later.”

(P-18)

“When the orthopedist referred me to the physical therapy department, I waited 2 months to make an appointment at the physical therapy department. After being examined by a physiatrist, I also waited for a long time to start the physiotherapy program. This waiting process made me very tired.”

(P-17)

“I couldn’t make an appointment with my physician because there were so many patients in the hospital. I had to wait 1 months. When my pain got worse during the waiting period, I used a lot of painkillers to relax.”

(P-19)

“When the medications did not help, I was able to start physiotherapy 3 months later because the physiotherapy unit was very busy. There were a lot many patients waiting to have physiotherapy. Therefore, I don’t think I had effective physical therapy. They don’t spare enough time for patients; they don’t inform patients about their disease.”

(P-20)

“Although I constantly try to make an appointment, I cannot make it. As I wait, my knee gets worse, even if I make an appointment, I cannot make another appointment with the same doctor, my treatment is constantly interrupted and I am fed up with this process.”

(P-1)

### 3.3. Theme 3: Noncompliance with Lifestyle Changes

The researchers observed that the patients had difficulty adapting to the lifestyle changes recommended by the physician during the conservative treatment process. In particular, older patients with comorbidities did not comply with lifestyle changes. Most of the patients stated that they were referred to a dietitian by their physicians because they were overweight. We observed that most of the patients lacked the willpower to lose weight. The patients also stated that they had difficulty in changing their eating habits to lose weight and in moving due to knee pain. One patient stated that she could not diet to lose weight, so she had to undergo stomach reduction surgery.

“My doctor told me that I should go to a dietitian and lose weight, I went to the dietitian, but I lose weight very slowly because I can’t walk.”

(P-14)

“I am overweight, but it is very difficult to lose it. I went to a dietician, but I can’t go on a diet because I like to eat, I can’t walk, I don’t know how to lose weight this way.”

(P-11)

“I was aware that I had to change my lifestyle, but I didn’t have the strength or willpower to do it. I could neither exercise nor go on a diet. I had to go to general surgery department to have stomach reduction surgery, and now my knee pain went away thanks to the weight loss.”

(P-17)

“I went to a dietician, I lost 3 kg, but as soon as I broke the diet a little, I gained 5 kg back. I’m so tired of dieting.”

(P-2)

## 4. Discussion

In our study, we interviewed 21 patients using the face-to-face interview method. In qualitative studies, the aim is to provide information without adhering to a specific structure, generally with small samples. These studies can have smaller sample sizes than quantitative studies, since they are thematic, versus statistical. The purpose of qualitative studies is to obtain in-depth information about the phenomenon/person examined and to create ideas for studies to be conducted in the future.

In the present study, we investigated the difficulties experienced by the patients with knee osteoarthritis during the conservative treatment process. One of the themes that emerged was the lack of information about conservative treatments. Most of the patients were not knowledgeable enough about their disease. In several other studies, the researchers also determined that patients had inadequate knowledge about knee osteoarthritis [19,22,25]. In a study conducted by Wallis et al. [25], some patients expressed their concern about structural changes in their joints and said that their physicians told them that the bones in their knees touched each other and that they would eventually need prosthetic surgery. Our patients’ descriptions of knee osteoarthritis, such as ‘calcification, osteoporosis and loss of fluid’, are consistent with the patients’ descriptions of their disease in other qualitative studies [25,26]. In several studies, it was determined that patients did not have detailed knowledge about their diseases [27] and that they wanted to have more detailed information about their treatments [19,20,22]. Patients wanted to know more about knee osteoarthritis, but they thought that physicians did not allocate enough time to provide detailed information about their disease [28]. In their study, Kamsan et al. [19] stated that many of the patients associated the development of knee osteoarthritis with factors such as aging, profession, inappropriate shoes, trauma, and being overweight. In several other studies, patients thought that knee osteoarthritis was associated with similar factors [29,30,31]. In a study conducted by Alami et al. [28], patients stated that knee osteoarthritis was an inevitable age-related disease, that not much could be done to change the stage of the disease, that treatments were of little help to cure the disease, and that physicians did not have many options to offer for treatment. We think that physicians should first answer patients’ questions clearly and concisely while they treat them. It seems very difficult to manage the conservative treatment process without eliminating the patient’s lack of information.

Another theme emerged in this study was “frequent physician change”. Patients’ constantly changing physicians makes conservative treatment difficult. During the conservative treatment process, patients’ trusting the physician and complying with the treatment recommended by the physician is of great importance. In a study conducted in Australia, the patients stated that receiving recommendations from a reliable physician facilitated the treatment process [20]. In the same study, they also stated that they trusted orthopedic surgeons in the early stages of their disease, and physiotherapists regarding exercise. Many factors affect patients’ trust in their physicians. According to a study, physicians having a special and individualized relationship with the patient makes the patient feel good and establish a sense of trust in the physician [28]. In the same study, the patients stated that easy accessibility of the physician and his being determined in the management of the disease made them perceive the physician as their own doctor. In the current study, on the contrary, the patients were confused due to different treatment recommendations made by physicians and therefore they did not trust them. In another study, the patients thought the physician’s age, education and reputation were related to his or her professional competence [28]. According to patients, having the feeling of trust in the physician is the most effective way to cope with the uncertainty of their medical situation. When a sense of trust is not established, patients’ treatment process takes longer, and it is hard to achieve effective treatment. In this case, it is important for the physician to allocate enough time to the patient, to provide clear information in a way the patient can easily understand, and to answer the questions to be asked by the patient to create a sense of trust [28]. Due to the health policies implemented in Turkey, it is impossible for physicians to devote enough time to their patients, which is one of the leading factors that prevent the establishment of a sense of trust. We think that further research should be conducted on the effect of the patient–physician trust relationship on the conservative treatment process.

After the researcher held interviews with the patients, he determined that the patients had difficulties in accessing healthcare services for the treatment of knee osteoarthritis. One of the difficulties they had was related to making an appointment with a physician for outpatient treatment. In another study, the patients stated that they waited for a long time to make an appointment for the treatment of knee osteoarthritis, especially in public hospitals [32]. In a study, the patients stated that waiting for a long time to see the doctor due to having difficulty in making an appointment disappointed them [33]. In the present study, the patients’ compliance with the treatment process was poor, especially among patients who had difficulty making an appointment with the physiotherapy unit. In another study, the patients were confused when they tried to make an appointment with the physiotherapy department [33]. They also stated that they waited 2 months to make an appointment, which hindered their treatment process [33].

In the present study, the patients stated that even if they were admitted to the physiotherapy unit after a long waiting period, they did not think that physiotherapy would be an effective and useful method. In their study, Hills and Kitchen [34] investigated patients’ satisfaction with physiotherapy, and they determined that the patients’ satisfaction with the clinical results of physiotherapy was poor. In another study, the patients who received adequate information and feedback about exercise performance from a physiotherapist before, during and after treatment complied with the treatment better [20]. In the same study, some of the patients perceived the physiotherapists’ not allocating enough time to them as an obstacle to their treatment. In the present study, the patients stated that physiotherapists did not care enough about them and did not inform them about the treatment adequately. Thus, the patients’ compliance with the physiotherapy process was negatively affected. The preparation of patient-specific physiotherapy programs can facilitate compliance with treatment. There is a need for more research on this issue.

The third theme emerged in the present study was “non-compliance with lifestyle changes”. Most of the patients participating in the present study stated that they were referred to dietitians and exercise programs by their physicians because they were obese. Of the patients, those who were referred to a dietitian to lose weight were not accustomed to dieting and had difficulty in complying with the diet list. Especially older patients stated that they had difficulty in changing their eating habits. In a study, the patients stated that they were aware that they were overweight, that they knew what foods they should eat to lose weight, and that losing weight would be beneficial for them [35]. However, being aware is not enough for patients. To lose weight, they must demonstrate their will. In a study conducted by Carmona-Terés et al. [35], one of the common beliefs among the patients was that losing weight was impossible. In the same study, especially older female patients stated that they attributed the impossibility of losing weight to menopause, age, comorbidities (such as diabetes, thyroid disease) and inactivity. In another study, the patients had the same concern about complying with nutritional recommendations [36]. In the present study, although the patients complied with the diet program recommended by the dietician, they could not lose weight because they could not comply with the exercise program sufficiently due to knee pain. Lowering body mass index is an important part of the conservative treatment process. For this purpose, diet and exercise programs that suit the patients’ current lifestyle and eating habits must be organized.

The inclusion of only patients in the study is a limitation of this study. Therefore, the results obtained from the research are valid only from the point of view of patients. We recommend that future studies should also include orthopedic surgeons, physical therapy and rehabilitation physicians, dietitians and physiotherapists who have a role in multidisciplinary management of patients and the conservative treatment process together.

## 5. Conclusions

In the present study, the following three main themes emerged regarding the difficulties experienced by patients with knee osteoarthritis during the conservative treatment process: lack of knowledge about conservative treatments, frequent change of physicians, and non-compliance with lifestyle changes. In conservative treatment, pharmacological and non-pharmacological treatment methods should be combined. In non-pharmacological treatment planning, patient education, weight control, physical activity and exercise programs should be considered as a whole. Therefore, in the conservative treatment process of patients with knee osteoarthritis, a multidisciplinary approach should be adopted, and orthopedic surgeons, physical therapy and rehabilitation physicians, dietitians and physiotherapists should be in harmony with the patient. During the multidisciplinary approach, a patient-specific treatment plan should be prepared, and the relevant departments should be contacted and included in the treatment process. The healthcare personnel who will take part in this process should be aware of the planning. Ensuring patients’ compliance with the conservative process is not only the physician’s responsibility but also everyone else’s in the team. Health personnel should provide patients with detailed information to eliminate questions they have during the conservative treatment process. In order to establish a trust relationship between the patient and the physician, the physician’s allocating enough time to the patient and including the patient in the treatment plan to ensure the patient’s compliance with the treatment is of importance. During the treatment process, it should be made easier for patients to make an appointment for the day they want to visit their doctor, and patients should be prevented from waiting for a long time without treatment. When patients want to visit the same physician for a second follow-up examination during the treatment process, they should be ensured that they can do so without an appointment.

## Figures and Tables

**Table 1:** Semi-structured interview questions.

1. What do you know about knee osteoarthritis? How would you describe it? How do you think your disease developed?
2. How do you think your disease will be treated? What treatment options other than surgery do you know?
3. Which physicians did you consult for the treatment of your disease? What treatments did the physicians you consulted recommend?
4. How was your communication with the physician during the treatment process? What do you think about the information given by the physician?
5. What do you think prevented you from keeping up with the treatment process? Why did you have difficulty keeping up with your treatment?

**Table 2:** Demographic data of the patients.

Participant (P)	Age	Sex	Educational Status	Profession	Duration of Having Symptoms (Years)	Side	KL Grade	BMI
P1	58	W	PSG	Homemaker	8	L	2	24.09
P2	61	W	Illiterate	Homemaker	10	R	3	28.34
P3	66	M	PSG	Retired	5	R	3	26.57
P4	63	M	PSG	Farmer	3	L	2	31.88
P5	67	M	PSG	Retired	7	L	3	30.11
P6	56	M	PSG	Farmer	5	L	2	35.55
P7	59	W	Illiterate	Homemaker	1	L	2	33.33
P8	62	M	PSG	Farmer	2	L	2	37.77
P9	65	W	Illiterate	Homemaker	8	R	3	32.18
P10	64	W	Illiterate	Homemaker	4	R	3	29.74
P11	61	M	PSG	Farmer	1	L	3	39.31
P12	57	W	Illiterate	Homemaker	3	R	2	29.71
P13	60	W	Illiterate	Homemaker	2	R	2	24.80
P14	63	M	PSG	Farmer	10	R	3	31.17
P15	66	M	PSG	Retired	9	R	3	28.86
P16	68	W	Illiterate	Homemaker	12	L	3	25.57
P17	61	W	Illiterate	Homemaker	5	R	2	38.53
P18	62	W	PSG	Farmer	8	R	2	28.84
P19	69	W	PSG	Homemaker	14	L	3	31.45
P20	70	W	Illiterate	Homemaker	13	L	3	30.62
P21	67	W	PSG	Homemaker	12	L	3	24.78

W = Women, M = Men, PSG = Primary School Graduate, L = Left, R = Right, KL grade = Kellgren and Lawrence’s grade, BMI = Body Mass Index.

**Table 3:** The themes and sub-themes.

Themes	Sub-Themes
1. Lack of knowledge about conservative treatments	-
2. Frequent physician change	2.1 Distrusting health personnel 2.2 Not being able to make an appointment
3. Noncompliance with lifestyle changes	-

## Data Availability

Research data are unavailable due to privacy and ethical restrictions of our patients.
